# Early Cardiovascular and Metabolic Benefits of rhGH Therapy in Adult Patients with Severe Growth Hormone Deficiency: Impact on Oxidative Stress Parameters

**DOI:** 10.3390/ijms26125434

**Published:** 2025-06-06

**Authors:** Maria Kościuszko, Angelika Buczyńska, Justyna Hryniewicka, Dorota Jankowska, Agnieszka Adamska, Katarzyna Siewko, Małgorzata Jacewicz-Święcka, Marcin Zaniuk, Adam Jacek Krętowski, Anna Popławska-Kita

**Affiliations:** 1 Department of Endocrinology, Diabetology and Internal Medicine, Medical University of Bialystok, 15-274 Bialystok, Poland; ak001@wp.pl (A.A.); katarzynasiewko@o2.pl (K.S.); malgorzata.jacewicz-swiecka@umb.edu.pl (M.J.-Ś.); marcin.zaniuk@gmail.com (M.Z.); adamkretowski@wp.pl (A.J.K.); annapoplawskakita@op.pl (A.P.-K.); 2 Clinical Research Center, Medical University of Bialystok, 15-274 Bialystok, Poland; angelika.buczynska@umb.edu.pl (A.B.); justyna.hryniewicka@umb.edu.pl (J.H.); 3 Department of Biostatistics and Medical Informatics, Medical University of Bialystok, 15-274 Bialystok, Poland; dorota.jankowska@umb.edu.pl

**Keywords:** growth hormone, growth hormone deficiency, endothelin-1, oxidative stress, asymmetric dimethyl arginine

## Abstract

It is hypothesized that growth hormone deficiency (GHD) is associated with increased oxidative stress (OS), contributing to elevated cardiovascular risk. This preliminary study evaluates changes in OS markers and cardiovascular biomarkers in 15 adult patients with severe GHD undergoing 12 months of recombinant human growth hormone (rhGH) therapy. IGF-1 concentrations increased significantly following 6 and 12 months of therapy (*p* = 0.0003 and *p* = 0.0001, respectively). These changes were accompanied by a significant decrease in endothelin-1 (ET-1) levels at 12 months (*p* = 0.007), as well as reductions in asymmetric dimethylarginine (ADMA) levels at both 6 and 12 months (*p* = 0.01 for each timepoint). Total oxidative capacity (TOC) decreased significantly after 6 months of therapy (*p* = 0.02), followed by a significant increase at 12 months (*p* = 0.04), whereas total antioxidant capacity (TAC) showed a significant increase at 12 months (*p* = 0.02). Tissue fat % showed significant reductions at 6 months (*p* = 0.006), suggesting early improvements in body composition. Correlation analyses indicated negative associations between IGF-1 and TOC (*p* < 0.006; R = −0.73), and positive associations with TAC (*p* < 0.001; R = 0.83). These findings suggest that rhGH therapy in adult patients with severe GHD reduces OS and cardiovascular risk through the modulation of biomarkers and improved body composition. This study explores the role of rhGH therapy in reducing cardiovascular risks in GHD, emphasizing the importance of individualized treatment approaches.

## 1. Introduction

Growth hormone (GH) significantly influences growth, metabolism, and overall health, with insulin-like growth factor I (IGF-1) acting as its key mediator. In adults, growth hormone deficiency (GHD) may either persist from childhood (CO-GHD) or develop in adulthood (AO-GHD), affecting approximately 2–3 individuals per 10,000 [1,2,3,4]. Pituitary somatotropin deficiency may result from congenital or genetic mutations in genes such as *POUF1*, *PROP-1*, *HESX-1*, *LHX-3*, and *LHX-4*, or from mutations causing isolated GHD and brain development disorders. Acquired GHD is most commonly caused by pituitary–hypothalamic tumors but can also result from Langerhans cell histiocytosis, head trauma, hydrocephalus, empty sella syndrome, or unidentified factors [5].

Recombinant human growth hormone (rhGH) therapy, introduced over six decades ago, has revolutionized GHD treatment, impacting growth and metabolic processes like lipid, carbohydrate, and protein metabolism [6,7,8,9]. GHD increases cardiovascular disease (CVD) risk and mortality, but rhGH therapy reduces cardiometabolic risk factors; improves lipid profiles and endothelial function; and decreases markers of inflammation, such as adipokines and oxidative stress (OS) [10,11,12]. OS contributes to CVD by damaging macromolecules and promoting atherosclerosis [12,13,14,15,16,17]. Molecules like endothelin-1 (ET-1), nitric oxide (NO), and IGF-1 are essential for vascular homeostasis. IGF-1 enhances NO synthesis, improving endothelial and cardiac function [18,19,20]. NO, a vasodilator, maintains vascular tone, but OS or elevated asymmetric dimethylarginine (ADMA) levels, which inhibit NO synthase, reduce NO bioavailability, leading to endothelial dysfunction, higher vascular resistance, and increased CVD risk [21,22,23]. In addition to cardiovascular effects, GHD leads to increased fat deposition, especially in visceral adipose tissue, contributing to obesity. This accumulation exacerbates insulin resistance (IR), impairing insulin sensitivity and GH function [24]. The visceral adiposity index (VAI) is a useful tool for assessing IR by analyzing lipid profiles to evaluate visceral fat distribution and its metabolic effects. Studies have shown strong links between VAI and higher CVD risk, and a negative correlation with tissue insulin sensitivity [25,26,27].

Although rhGH therapy has been effective in reducing metabolic and cardiovascular risks associated with GHD, the exact mechanisms behind these effects are not fully understood [12]. Specifically, the roles of ET-1, ADMA, NO, and OS in relation to rhGH therapy, as well as their interactions with lipid profiles, body composition, and IR, need further investigation. Identifying reliable biochemical markers is essential for developing personalized treatment strategies and optimizing the monitoring of rhGH therapy’s impact on cardiovascular risk and metabolic health in GHD patients. Additionally, given that GH and IGF-1 play essential roles in calcium (Ca) homeostasis by influencing both bone metabolism and cardiovascular health, evaluating calcium levels provides valuable insight into the metabolic consequences of GHD [28].

The aim of this study was to evaluate the impact of rhGH therapy on cardiovascular and metabolic parameters in patients with GHD. Key biomarkers, including ET-1, ADMA, and NO, as well as OS parameters such as total oxidative capacity (TOC) and total antioxidant capacity (TAC), were analyzed. Additionally, lipid profiles, body composition, and IR were assessed. The findings aim to provide insights into the early effects of rhGH therapy, with the potential to incorporate these biomarkers into routine clinical practice to improve treatment outcomes and reduce long-term cardiovascular risk in GHD patients.

## 2. Results

### 2.1. Biochemical Analysis

#### 2.1.1. IGF-1 and Ca Measurements

**Figure 1:**
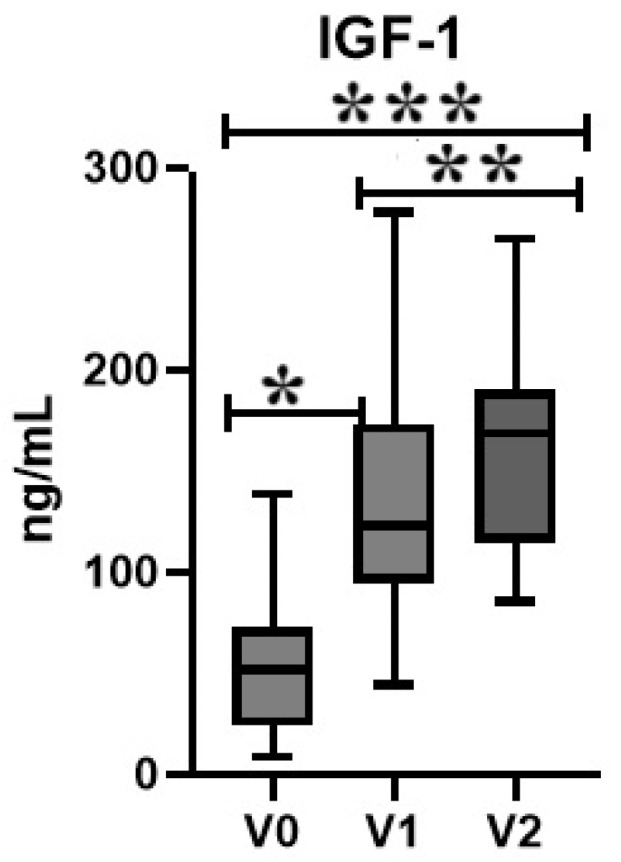
Changes in serum insulin-like growth factor type 1 (IGF-1) concentrations during rhGH therapy in patients with growth hormone deficiency (GHD). A significant increase in IGF-1 levels was observed after 6 months (V1) (*p* = 0.003 *) and maintained after 12 months (V2) of treatment compared to baseline (V0) (*p* = 0.0001 ***). However, no statistically significant difference was found between the 6- and 12-month timepoints (*p* = 0.15 **). Values are shown as box plots with median, interquartile range, and full range.

Decreased IGF-1 levels were defined as values below −2 standard deviation scores (SDS) from the age- and sex-adjusted reference mean, in accordance with current clinical guidelines. At study entry, 13 out of 15 patients (86.7%) had IGF-1 concentrations below this cut-off. Moreover, in the studied group of patients, significantly higher IGF-1 concentrations were observed after 6 and 12 months of therapy compared to baseline values (*p* = 0.0003 and *p* = 0.0001, respectively). We did not observe statistically significant differences in the concentrations of IGF-1 at 12 months of therapy compared to 6 months (*p* = 0.15). Moreover, we noted significantly higher concentrations of Ca after 12 months compared to baseline (*p* = 0.01) (Table 1, Figure 1).

#### 2.1.2. Endothelin-1, Asymmetric Dimethylarginine, and Oxidative Stress

**Figure 2:**
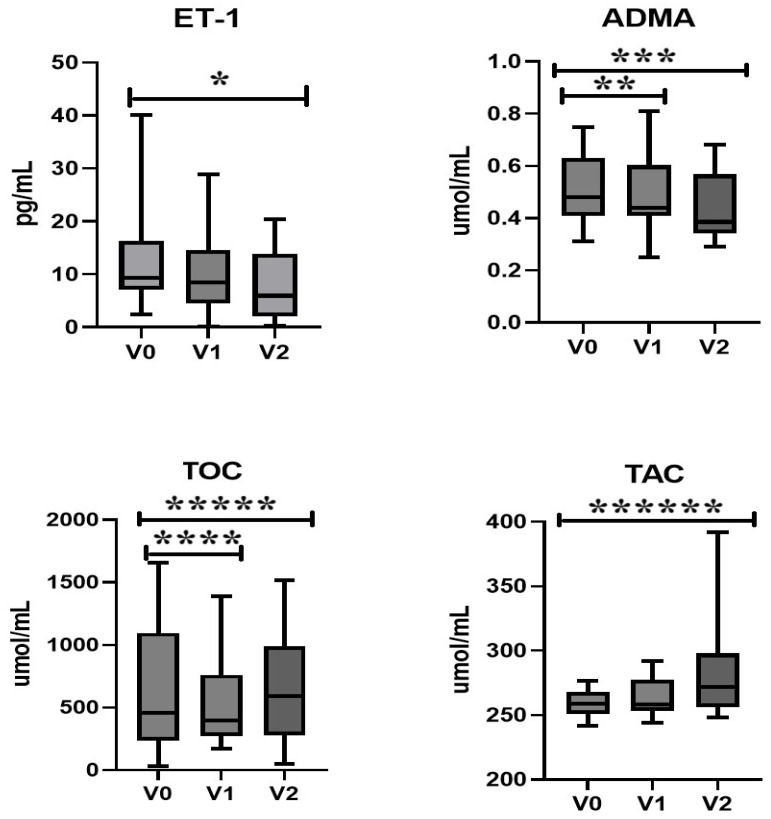
Effect of rhGH therapy on endothelin-1 (**ET-1**), asymmetric dimethylarginine (**ADMA**), total oxidant capacity (**TOC**), and total antioxidant capacity (**TAC**) levels in patients with growth hormone deficiency (GHD). After 12 months of treatment (V2), ET-1 concentrations were significantly reduced compared to baseline (V0) (*p* = 0.007 *), while no significant change was seen at 6 months (V1). ADMA levels showed a significant decrease as early as V1, which persisted through V2 (*p* = 0.01) **, ***). TOC levels were significantly lower at both V1 and V2 (*p* = 0.02 ****, and *p* = 0.04 *****, respectively), indicating reduced oxidative stress. In contrast, TAC levels showed a significant increase at V2 (*p* = 0.02 ******), suggesting improved antioxidant capacity. Box plots represent median values with interquartile ranges and full data spread.

Statistically significantly lower concentrations of ET-1 after 12 months of therapy were observed compared to the baseline value (*p* = 0.007). We did not observe significant differences in ET-1 concentrations after 6 months of therapy. Statistically significantly lower concentrations of ADMA after 6 and 12 months of therapy were observed compared to the baseline value (*p* = 0.01). Significantly lower concentrations of TOC were observed after 6 months compared to the baseline, followed by an increase after 12 months (*p* = 0.02 and *p* = 0.04, respectively). Additionally, TAC concentrations were significantly higher after 12 months compared to the baseline (*p* = 0.02) (Table 1, Figure 2).

#### 2.1.3. Nitric Oxide and Lipid Profile

No statistically significant changes in the concentration of NO or the lipid profile were observed (Table 1).

### 2.2. DXA and Body Composition

At baseline, 4 out of 15 patients (26.6%) met the WHO criteria for obesity (BMI ≥ 30 kg/m^2^). In the GHD group, we observed statistically significant differences in fat tissue % after 6 and 12 months of therapy compared to the baseline value (*p* = 0.006 and *p* = 0.04, respectively). Following an initial reduction in fat tissue percentage during the first 6 months of therapy, a subsequent increase was noted by the 12th month of treatment. However, no statistically significant differences were observed in total mass, fat tissue (g), lean mass, bone mineral content (BMC), L1-L4 density, and femoral neck density. Additionally, no statistically significant changes in the VAI index were found during the course of the therapy (Table 2).

### 2.3. Correlations

In the study group, we observed a statistically significant negative correlation between IGF-1 concentration and TOC capacity after 6 and 12 months of therapy (*p* < 0.006; R = −0.73, *p* < 0.01, R = −0.69, respectively). Moreover, we noted a positive correlation between IGF-1 and TAC capacity after 6 and 12 months (*p* < 0.001; R = 0.83, *p* < 0.01; R = 0.69, respectively). Moreover, in the GHD group, IGF-1 demonstrated a moderate negative correlation with ADMA (*p* < 0.01; R = −0.65) and NO (*p* < 0.03, R = −0.67) after 12 months of therapy. Furthermore, in the treated group, we initially observed a statistically significant negative correlation between IGF-1 and NT-pro-BNP concentration (*p* < 0.02; R = −0.62). After 6 months, we noted a positive correlation between TOC capacity and NT-pro-BNP concentration (*p* < 0.04; R = 0.56). Additionally, we noted a moderate negative correlation between TOC and lean mass initially (*p* < 0.03; R = −0.52). Moreover, HDL concentration was noted to have a positive correlation with TOC initially (*p* < 0.04; R = 0.49) and a negative correlation with TAC after 12 months of treatment (*p* < 0.01; R = −0.72). In the conducted observation, a statistically significant correlation between NO concentration and baseline fat tissue % was demonstrated (*p* < 0.04; R = 0.51). Moreover, NT-pro-BNP value showed a statistically significant negative correlation with total cholesterol and LDL levels after 6 months of therapy (*p* < 0.01; R = −0.70, *p* < 0.01; R = −0.84, respectively). Furthermore, a statistically significant negative correlation was demonstrated at baseline between ET-1 concentration and total mass, tissue mass, lean mass, BMC, BMD, and Ca. Moreover, a negative correlation was also observed after 12 months between ET-1 concentration and lean mass (*p* < 0.001; R = −0.81) and BMC (*p* < 0.0001; R = −0.84). ADMA initially correlated positively with total mass, tissue mass, lean mass, and fat tissue. Moreover, a significant positive correlation between ADMA and BMI was observed after 12 months of therapy (*p* < 0.02; R = −0.64). Furthermore, a significant negative correlation between VAI and HDL levels at baseline was observed (*p* = 0.002, R = −0.77). In contrast, a strong positive correlation between VAI and TG (*p* = 0.01, R = 0.68) at baseline was also noted. All correlations are presented in Table 3.

## 3. Discussion

Untreated GHD is associated with increased mortality, particularly from CVD [29]. However, rhGH therapy has been shown to improve cardiometabolic risk factors, including lipid abnormalities, endothelial dysfunction, atherosclerosis, and cardiovascular inflammation markers such as adipokines and OS [30,31]. Additionally, GHD patients typically exhibit increased fat mass, mainly visceral fat, and reduced lean body mass, which contribute to higher cardiovascular risk [32,33]. Chronic low-grade inflammation, linked to fat tissue and marked by elevated inflammatory markers and cytokines, leads to the secretion of reactive oxygen species (ROS) by adipocytes, which exacerbates the condition and increases OS due to an imbalance between ROS production and the body’s antioxidant defenses, ultimately raising the risk of CVD [34,35,36,37]. Additionally, high ADMA levels, a potent inhibitor of NO synthase, impair vascular endothelial function and are associated with increased vascular resistance, higher blood pressure, and atherosclerosis [22,38,39].

IGF-1 acts as an anti-inflammatory agent by modulating immune cell activity and reducing the production of pro-inflammatory cytokines, such as interleukin-6 (IL-6) and tumor necrosis factor-alpha (TNF-α) [40]. This helps to reduce vascular inflammation, lowering the risk of vessel damage and the progression of atherosclerosis. Additionally, IGF-1 enhances the body’s antioxidant mechanisms, protecting the endothelium from ROS. By increasing the activity of antioxidant enzymes like superoxide dismutase (SOD), IGF-1 mitigates OS, reducing vascular damage and the risk of CVD linked to excessive free radical production [41]. Previous studies have described the potential impact of obesity—along with associated hepatic steatosis, decreased ghrelin levels, and hyperinsulinemia affecting IGF-1 binding protein (IGF-BP) concentrations—on the reduction of IGF-1 levels, particularly in individuals with a BMI in the range of 30–35 kg/m^2^ [42]. On the other hand, some authors have also reported conflicting results, including normal or even elevated IGF-1 levels in obese patients [43]. In the study group, 26.6% of participants were classified as obese. Therefore, in accordance with current recommendations, the assessment of IGF-1 levels in our study accounted for a standard deviation score below –2 SD. Following rhGH therapy in the study group, a clinically significant increase in IGF-1 concentration was observed after 6 and 12 months compared to the baseline value. We did not observe statistically significant differences in the concentrations of IGF-1 at 6 months of therapy compared to 12 months (*p* = 0.15). The statistically significant increase in IGF-1 concentration observed during the therapy was attributed to the individual adjustment of rhGH dosage aimed at achieving optimal treatment outcomes. In our study, consistent with findings from other researchers, we observed a statistically significant increase in IGF-1 levels after just 6 months of rhGH therapy [44,45]. The result of our observation suggests a potential indirect reduction in cardiovascular risk after just 6 months of therapy.

Emerging evidence suggests that ET-1 and related peptides play a significant prognostic role in coronary artery disease, hypertension, and heart failure [46,47,48]. Furthermore, ET-1 has been investigated as both a predictor and prognostic marker in cardiovascular events, often associated with cardiac remodeling, such as increased left atrial diameter and left ventricular mass [49]. Additionally, NO functions as a crucial vasodilator, promoting blood vessel dilation by relaxing smooth muscle cells in the vessel walls [50]. The balance between ET-1 and NO is crucial for regulating vascular tone and maintaining vascular stability. Any disruption in this balance can lead to various CVD [51,52]. Additionally, there are reports indicating that intravenously administered ET-1 in healthy men inhibits the increase in GH levels stimulated by growth hormone-releasing hormone [53]. In this study, ET-1 levels significantly decreased after 12 months of rhGH therapy, suggesting reduced cardiovascular risk. However, the lack of significant changes at 6 months may reflect the individualized dosage adjustments required for optimal outcomes.

ADMA arises from the irreversible methylation of arginine residues and acts as an independent risk factor for CVD [54,55]. ADMA has the ability to act as a competitive inhibitor of NO synthase enzymes and contributes to the development and advancement of microvascular complications by impacting endothelial cell function, OS-induced damage, inflammation, and fibrosis [56,57]. In the examined GHD group, we observed a statistically significant lower concentration of ADMA after just 6 months, with a declining trend persisting at the 12-month mark of therapy compared to the baseline value (*p* = 0.01 and *p* = 0.01, respectively). These findings differ from previous studies, such as those by Improda et al., reporting ADMA reductions only after 12 months of therapy [58].

OS plays a pivotal role in the pathogenesis of CVD, driven by an imbalance between ROS production and antioxidant defenses [59,60,61,62]. Patients with GHD are characterized by elevated OS, as demonstrated by Mancini et al. [63,64]. In our study group, TOC concentrations exhibited a statistically significant reduction after 6 months of rhGH therapy compared to baseline, followed by a significant increase at 12 months. Although no overt clinical manifestations were observed, the elevation in TOC at 12 months may reflect a shift toward a pro-oxidative state. These findings raise concerns regarding the potential long-term impact of rhGH therapy on OS homeostasis and underscore the need for further investigation. Furthermore, in the GHD group, IGF-1 demonstrated a negative correlation with TOC, as well as with NT-pro-BNP concentration initially. IGF-1 can influence this process through various mechanisms, including the regulation of antioxidant enzyme expression, such as SOD and catalase, which help mitigate OS by neutralizing ROS [41,65]. Concurrently, GHD can decrease the activity of the antioxidant system, impairing the ability to neutralize ROS [66]. This decline may be due to reduced levels of antioxidants or diminished antioxidant effectiveness. In the GHD group, IGF-1 initially showed a moderate positive correlation with TAC. Our findings align with those of Mohn et al., who reported an increase in TAC after one year of rhGH therapy [67]. These findings suggest that rhGH treatment, and consequently the normalization of IGF-1 levels, may have a beneficial effect in reducing OS by enhancing antioxidant levels, potentially lowering cardiovascular risk.

The relationship between ADMA and TAC is complex. ADMA-induced endothelial dysfunction and OS can impair TAC by reducing antioxidant enzyme activity and depleting non-enzymatic antioxidants. Conversely, antioxidants mitigate ADMA’s effects by scavenging ROS and restoring endothelial function [39,68,69]. In our study, a positive correlation was observed between ADMA and TAC. Additionally, IGF-1 levels showed a moderate negative correlation with ADMA after 12 months of rhGH therapy. A positive correlation was also noted between IGF-1 and HDL and between TOC and HDL.

HDL particles contribute to reducing OS, which supports their role in preventing atherosclerosis [70]. HDL contains enzymes such as paraoxonase-1 (PON1), which breaks down lipid peroxides, thereby protecting lipids from oxidation [71,72]. However, HDL levels and their functional properties may differ significantly. Oxidative changes to HDL molecules can impair their functionality, potentially leading to pro-inflammatory and pro-atherogenic effects [71]. Studies show an inverse correlation between HDL levels and TOC, as higher HDL levels are typically associated with reduced OS [73]. However, in pathological states such as atherosclerosis or diabetes, this correlation may be disrupted [70,71,72,73,74]. In our study, HDL positively correlated with TOC at baseline and negatively correlated with TAC after 12 months of rhGH therapy. The obtained research results suggest that in the examined group of patients with GHD in a state of elevated OS due to GHD, the observed increased levels of HDL do not fulfill their protective function. Moreover, the findings of our study suggest that rhGH substitution therapy may modulate the oxidative balance of the body. Specifically, a significant increase in TAC was observed after 12 months of rhGH administration, indicating an improvement in the overall antioxidant status. This enhancement in TAC may be associated with increased levels of HDL cholesterol. Nevertheless, the observed relationships are preliminary, and we recommend further research to validate these findings.

The relationship between NO and GHD is complex and multifaceted, given their significant roles in diverse physiological functions. In individuals with GHD, alterations in NO levels or pathways may influence vascular function, potentially heightening cardiovascular risk. Conversely, GHD can result in decreased NO production, affecting endothelial function [75,76]. In our study, a statistically significant negative correlation between IGF-1 and NO was observed after 12 months of treatment. Our findings suggest that rhGH replacement therapy in GHD patients has improved endothelial function and NO levels, potentially reducing cardiovascular risk.

GH plays a key role in altering body tissue structure and composition, primarily through lipolysis and protein synthesis, with an anti-natriuretic function. It enhances lipolytic activity and sensitivity to hormones like adrenaline and testosterone, aiding fat reduction during rhGH therapy [77,78]. GH also stimulates protein synthesis [79]. In AO-GHD, there is increased fat tissue, especially visceral fat, and decreased fat-free mass, negatively impacting health and increasing cardiovascular risk [80]. rhGH therapy improves body composition by reducing fat mass and increasing muscle and fat-free mass, especially after 12 months [81]. Changes in fat and fat-free mass occur within the first 6 months and continue throughout treatment, with Postma et al. noting a slight increase in fat tissue after long-term rhGH use, though total fat mass remained lower compared to baseline after 4 years [82]. Overall, during rhGH treatment, body mass generally remains stable, as gains in fat-free mass are offset by reductions in fat tissue mass, as evidenced in our study, which showed no significant changes in weight after 6 and 12 months of therapy. The beneficial effects of GH on total body fat and its distribution have been examined in our study by means of DXA. In the examined group of patients, we observed a statistically significant decrease in fat tissue % content after just 6 months of rhGH replacement therapy, consistent with previous observations (*p* = 0.006). In contrast to the current research, we observed a subsequent increase in fat mass percentage by the 12th month of treatment, practically returning to baseline values. Furthermore, ET-1 displayed a moderate negative correlation with tissue mass, BMC, total mass, and lean mass. The observed correlations suggest that rhGH treatment, through increased IGF-1 levels and its positive association with tissue, total, and lean mass, leads to a reduction in cardiovascular risk, reflected by decreased ET-1 levels. Previous studies indicate that the increase in BMD due to rhGH therapy occurs after about 18 months, initially preceded by a decrease in BMD due to increased bone remodeling [83]. This may explain the lack of statistically significant changes in BMD in our study group. Nonetheless, we found a negative correlation between BMD and ET-1 concentration (*p* < 0.01; R = −0.61), suggesting a direct relationship between ET-1, cardiovascular risk, and bone metabolism.

GH also exerts a significant influence on glucose metabolism. It stimulates lipolysis, increasing the availability of free fatty acids as an energy substrate, and modulates insulin sensitivity indirectly through the action of IGF-1 [84]. Garmes et al. highlighted the intricate role of insulin signaling across the spectrum of GHD, emphasizing the interplay between GH and insulin sensitivity [85]. Their findings align with the need for tailored rhGH therapy to optimize metabolic outcomes in GHD individuals. Similarly, Fowelin et al. demonstrated that rhGH therapy improves insulin sensitivity and glucose metabolism in AO-GHD, highlighting its potential to restore metabolic balance [86]. Qiu et al. further explored the reciprocal influence of insulin on GH secretion and signaling, suggesting that IR can disrupt GH pathways [87]. Previous observations underscored IR as a critical cardiovascular risk factor, emphasizing its role in metabolic syndrome and CVD [88,89]. These studies support our findings on the metabolic benefits of rhGH therapy, particularly in reducing IR and the associated cardiovascular risks in GHD patients. Dong et al. examined GH’s role in diabetes pathogenesis, showing how altered GH signaling contributes to glucose metabolism dysregulation and IR [90]. This underscores GH’s complex role in metabolic balance and diabetes development. Similarly, Kim and Park discussed how GH affects glucose metabolism and IR, noting its potential to impair insulin sensitivity while promoting lipolysis and glucose production [91]. The limitations of the study include variability in patient cohorts, rhGH dosages, comorbidities, and the short duration of the observation period, all of which may have influenced the results. Key limitations also include the small sample size, which limits statistical power, and the possibility of bias despite the applied precautions. Moreover, due to the characteristics of the study population (with a maximum participant age of 60 years), the results may not be fully generalizable to older patients with GHD. Additionally, the benefits of rhGH therapy in elderly individuals may have a reduced impact on body composition [92]. Long-term studies focused on glucose homeostasis are necessary to better understand the metabolic impact of rhGH therapy in GHD.

## 4. Materials and Methods

### 4.1. Studied Population

The study, conducted at the Department of Endocrinology, Diabetology, and Internal Medicine, Medical University of Bialystok, Poland (grant APK.002.393.2021), involved 15 participants (4 females, 11 males) aged 18–60, all diagnosed with GHD. Severe GHD was diagnosed based on clinical symptoms, low IGF-1 levels, and GH secretion below 3 ng/mL in hypoglycemic tests using insulin and/or glucagon, in accordance with recommendations after correcting cortisol, thyroxine, and sex steroid deficiencies. GHD was diagnosed de novo in three patients during adulthood. In contrast, the other patients had been treated with rhGH during childhood, with therapy discontinuation occurring between one and twenty years prior. Fourteen patients had multiple pituitary hormone deficiencies, and one had isolated GH deficiency, confirmed by performing two GH stimulation tests (Table 4). The patients did not have a history of cardiovascular events or a current diagnosis of CVD, nor were they taking medications that could affect cardiovascular risk, such as statins, ezetimibe, or antiplatelet therapy. According to the World Health Organization (WHO) guidelines, patients were advised to engage in at least 150 min of moderate physical activity per week, spread over several days, or at least 75 min of vigorous physical activity per week, also spread over several days, or an equivalent combination of moderate and vigorous activity with a 2:1 ratio (e.g., 75 min of vigorous + 150 min of moderate activity). Obese patients were advised to follow a calorie-reduction diet with a daily energy deficit of 500–1000 kcal. Two male patients aged 18 and 25 years had a history of type 1 diabetes diagnosed 6 months prior; they were treated with intensive functional insulin therapy using human insulin analogs, achieving normal metabolic control (HbA1c < 6.5%). Exclusion criteria included severe general condition, uncontrolled metabolic diabetes (HbA1c > 7%), pre- or proliferative diabetic retinopathy, pregnancy, and a history of cancer. Patients started rhGH therapy at doses of 0.2 mg/day for males and 0.3 mg/day for females, with adjustments based on IGF-1 levels. Average daily doses were 0.5 mg/day for females and 0.4 mg/day for males. The administration of rhGH substitution therapy in personalized dosing regimens was not associated with any observed adverse effects. Anthropometric measurements, including height and weight, were performed using standardized instruments. Body mass index (BMI) was calculated by dividing body weight (kg) by height squared (m^2^). IR was estimated using the VAI, calculated differently for men and women as follows:

For males: VAI = [WC/(39.68 + (1.88 × BMI))] × (TG/1.03) × (1.31/HDL)(1)

For females: VAI = [WC/(36.58 + (1.89 × BMI))] × (TG/0.81) × (1.52/HDL)

Abbreviations used in the equations:

WC—waist circumference (cm)

BMI—body mass index (kg/m^2^)

TG—triglycerides (mmol/L)

HDL—high-density lipoprotein cholesterol (mmol/L)

Bone mineral density and body composition were assessed using dual-energy X-ray absorptiometry (DXA). The patients were non-smokers, did not abuse alcohol, and had no other conditions affecting peripheral OS. These data were collected from medical history, physical examination, and patient records. Venous blood samples (5.5 mL) were taken after fasting and centrifuged, and the serum was stored at −80 °C.

### 4.2. Biochemical Measurement

Measurements of TOC, TAC, NO, IGF-1, ET-1, and ADMA levels were taken prior to the commencement of the therapy (V0), at the 6-month mark (V1), and at the conclusion of the 12-month period (V2). Serum IGF-1 concentrations were measured using a chemiluminescent immunoassay (CLIA) on the Cobas e411 analyzer (Roche Diagnostics, Mannheim, Germany). To allow for comparisons accounting for physiological variability related to age and sex, IGF-1 values were converted to standard deviation scores (SDSs). The IGF-1 SDS was calculated as the number of standard deviations from the mean of an age- and sex-adjusted reference population, based on data provided by the assay manufacturer (Roche Diagnostics). The reference data were derived from a large population of healthy individuals and accounted for the known log-normal distribution of IGF-1 concentrations. A value below -2 SDS was considered significantly decreased. To assess oxidative status, the study relied on the quantification of TOC and TAC. The TOC status was determined through a photometric immunodiagnostic assay employing the PerOx (TOC/TAC) kit sourced from Immunodiagnostic KC 5100 and Immunodiagnostic KC 5200 (Immunodiagnostic Systems GmbH, Frankfurt, Germany). The assessment of TAC was conducted using the ImAnOx® assay (TAC, Antioxidative Capacity) (Immunodiagnostic Systems GmbH, Frankfurt, Germany), based on a colorimetric method. For the quantitative determination of ADMA in serum, the ADMA Xpress ELISA kit (K 7890, Immunodiagnostic Systems GmbH, Frankfurt, Germany) was used. NO was determined using a colorimetric method with the Colorimetric Assay Kit E-BC-K035-H (Elabscience Biotechnology Inc., Houston, TX, USA). Moreover, ET-1 was tested using the ELISA Kit E-EL-H0064 (Elabscience Biotechnology Inc., Houston, TX, USA). The ECLIA method was utilized to assay concentrations of N-terminal pro-brain natriuretic peptide (NT-pro-BNP) in serum using Elecsys proBNP II (Roche Diagnostics, 09315268190, Cobas e411 analyzer, Mannheim, Germany).

### 4.3. Statistical Analysis

Statistical analyses were performed using GraphPad Prism 9.0 software (GraphPad Software, San Diego, CA, USA) Data distribution was assessed with the Shapiro–Wilk test, indicating non-normal distribution. Consequently, nonparametric tests, including the Mann–Whitney (**) and Kruskal–Wallis (*) tests, were used for inter-group comparisons. Statistical significance was set at *p* < 0.05.

Data in the table are expressed as the median, along with the minimum and maximum values (MIN–MAX range). Spearman correlation analysis was conducted to evaluate relationships between parameters. Odds ratios (ORs) and logistic regressions were computed using GraphPad Prism v. 9.0. Statistical analysis involved repeated-measures ANOVA and post hoc tests with Bonferroni corrections.

### 4.4. Dual-Energy X-Ray Absorptiometry and Body Composition

The DXA method was employed to assess body composition using the medical body analyzer Hologic (USA). This device enables the measurement of bone mineral density, bone mineral content (BMC), body mass, total body water (TBW), fat mass, lean mass, and BMI.

## 5. Conclusions

This study provides evidence for the significant and early therapeutic effects of individualized rhGH therapy in adult patients with severe GHD. Notable improvements in cardiovascular and metabolic biomarkers were observed as early as 6 months following treatment initiation. A significant decrease in ET-1 concentrations was evident after 6 months, whereas reductions in ADMA were observed at 12 months. TOC levels decreased after 6 months of therapy but subsequently increased at the 12-month mark. Concurrently, TAC and IGF-1 levels showed significant increases over the treatment period. These findings collectively suggest a reduction in cardiovascular risk, improved metabolic homeostasis, and favorable modifications in body composition. The observed early changes underscore the clinical utility of rhGH therapy in mitigating long-term health risks associated with severe GHD in adult patients. Routine monitoring of relevant biomarkers, including ET-1, ADMA, and OS parameters, may enhance therapeutic precision and aid in identifying patients most likely to benefit from such treatment. Further studies with larger cohorts and extended follow-up are warranted to confirm these findings and refine therapeutic strategies.

## Figures and Tables

**Table 1:** Investigated biochemical parameters in the studied group.

Parameters		GHD Group (n = 15)			
	V0	V1	V2	*p* Value (V0 vs. V1)	*p* * Value (V0 vs. V2)
**IGF-1 (ng/mL)**	47.07 (8.57–138.8)	122.8 (44.1–278.1)	155.1 (36.04–265.1)	**0.0003**	**0.0001**
**ET-1 (pg/mL)**	8.67 (0.18–40.09)	8.4 (0.03–28.89)	5.93 (0.18–20.44)	0.24	**0.007**
**ADMA (umol/mL)**	0.5 (0.31–0.75)	0.43 (0.25–0.67)	0.38 (0.29–0.59)	**0.01**	**0.01**
**NO (umol/mL)**	23.54 (7.57–58.52)	30.79 (5.63–55.94)	31.11 (7.57–82.06)	1.0	0.73
**TAC** **(umol/L)**	258.6 (241.8–276.7)	258.3 (244.3–291.9)	271.1 (248.4–392.1)	0.22	**0.02**
**TOC** **(umol/L)**	457.3 (32.12–1655)	394.4 (171.9–1391)	589.5 (47.24–1514)	**0.02**	**0.04**
**Cholesterol** **(mg/dL)**	201 (114–302)	188 (87–296)	199 (114–295)	0.28	0.69
**LDL** **(mg/dL)**	126 (65–219)	121.5 (48–173)	131 (58–216)	0.85	0.20
**HDL** **(mg/dL)**	43 (24–85)	45 (26–76)	50 (27–80)	0.41	0.20
**TG** **(mg/dL)**	120 (51–684)	125 (55–259)	120.5 (45–326)	0.91	0.67
**NT-pro-BNP (pg/mL)**	45.13 (10–2025)	35.71 (10–1546)	27.16 (10–1325)	0.38	0.23
**Ca (mmol/L)**	2.31 (2.03–2.8)	2.32 (2.02–2.44)	2,37 (2.17–3.02)	0.18	**0.01**
**Glucose** **(mg/dL)**	89 (80–180)	90 (75–172)	86 (75–147)	0.19	0.15
**VAI**	4.31 (1.56–11.77)	4.23 (1.58–7.03)	3.58 (1.34–9.05)	0.59	0.69

Abbreviations: **GHD**: growth hormone deficiency; **IGF-1**: insulin-like growth factor type 1; **ET-1**: endothelin 1; **ADMA**: asymmetric dimethylarginine; **NO**: nitric oxide; **TAC**: total antioxidant capacity; **TOC**: total oxidant capacity; **LDL:** low-density lipoprotein; **HDL**: high-density lipoprotein; **TG**: triglycerides; **NT-pro-BNP**: N-terminal fragment of the pro brain natriuretic peptide **Ca**: calcium; VAI: visceral adiposity index, * *p* value comparing baseline (V0) and 12-month follow-up (V2) using appropriate non-parametric tests.

**Table 2:** Investigated bioimpedance parameters in the studied group.

Parameters		GHD Group (n = 15)			
	V0	V1	V2	*p* Value (V0 vs. V1)	*p* * Value (V0 vs. V2)
**Total mass (kg)**	78.6 (39.6–167.3)	85.8 (67.6–122.3)	78.0 (62.3–156)	0.72	0.51
**Tissue fat %**	37.5 (27.4–50.4)	36.3 (30.6–48.8)	38.4 (26.7–48.7)	**0.006**	**0.04**
**Tissue mass (g)**	76,222 (37,887–163,689)	79,671 (65,189–119,236)	73,551 (15,232–113,051)	0.19	0.11
**Fat tissue (g)**	28,434 (13,891–82,462)	30,931 (19,970–52,232)	29,937 (16,939–67,385)	0.23	0.08
**Lean mass (g)**	48,646 (23,996–81,228)	52,144 (36,852–73,131)	45,550 (36,530–84,977)	0.08	0.49
**BMC (g)**	2547 (1261–3778)	2637 (2149–3829)	2568 (1770–3650)	0.77	0.42
**L1-L4 BMD (g/cm^2^)**	1.09 (0.8–1.6)	1.08 (0.9–1.6)	1.1 (0.9–1.5)	1.0	0.73
**L1-L4 T score**	−1.1 (−3.4–3.2)	−1.0 (−2.1–2.8)	−0.3 (−2.0–2.4)	0.56	0.17
**L1-L4 Z score**	−1.1 (−3.7–3.0)	−0.7 (−2.3–2.7)	−0.9 (−2.3–2.0)	0.20	0.73
**Femoral neck BMD**	0.95 (0.7–1.4)	0.97 (0.78–1,4)	0.96 (0.77–1.5)	0.30	0.29
**Femoral neck T score**	−0.8 (−2.1–2.3)	−0.7 (−1.9–2.0)	−0.6 (−1.9–2.5)	0.58	0.79
**Femoral neck Z score**	−0.9 (−2.2–2.0)	−1.1 (−2.2–1.6)	−1.0 (−2.0–2.3)	0.59	0.72

Abbreviations: **GHD**: growth hormone deficiency; **BMC**: bone mineral content; **BMD**: bone mineral density; “−“: minus; * *p* value comparing baseline (V0) and 12-month follow-up (V2) using appropriate non-parametric tests.

**Table 3:** Spearman’s correlation coefficients between OS parameters and other metabolic markers in the studied group initially and during the treatment.

Parameters	V0	V1	V2
**IGF-1 vs. TOC**	***p *** **< 0.006; R = −0.73**	NS	***p *** **< 0.01; R = −0.69**
**IGF-1 vs. TAC**	***p *** **< 0.001; R = 0.83**	NS	***p *** **< 0.01; R = 0.69**
**IGF -1 vs. ADMA**	NS	NS	***p* < 0.01; R = −0.65**
**IGF-1 vs. NO**	NS	NS	***p* < 0.03; R = −0.67**
**IGF-1 vs. NT-pro-BNP**	***p *** **< 0.02; R = −0.62**	NS	NS
**TOC vs. NT-pro-BNP**	NS	***p* < 0.04; R = 0.56**	NS
**TOC vs. Lean mass**	***p *** **< 0.035; R = −0.52**	NS	NS
**TOC vs. HDL**	***p *** **< 0.04; R = 0.49**	**NS**	**NS**
**TAC vs. HDL**	NS	NS	***p *** **< 0.01; R = −0.72**
**NO vs. Fat tissue %**	***p *** **< 0.04; R = 0.51**	NS	NS
**NO vs. TG**	NS	NS	***p* < 0.004; R = 0.67**
**NT-pro-BNP vs. cholesterol**	NS	***p* < 0.01; R = −0.70**	NS
**NT-pro-BNP vs. LDL**	NS	***p* < 0.001; R = −0.84**	NS
**ET-1 vs. Total mass**	***p *** **< 0.05; R = −0.53**	NS	NS
**ET-1 vs. Tissue mass**	***p *** **< 0.05; R = −0.51**	NS	NS
**ET-1 vs. Lean mass**	***p *** **< 0.05; R = −0.55**	NS	***p* < 0.001; R = −0.81**
**ET-1 vs. BMC**	***p *** **< 0.01; R = −0.51**	NS	***p* < 0.0001; R = −0.84**
**ET-1 vs. BMD**	***p *** **< 0.01; R = −0.61**	NS	NS
**ET-1 vs. Ca**	***p *** **< 0.03; R = −0.65**	NS	NS
**ADMA vs. Total mass (kg)**	***p *** **< 0.02; R = 0.54**	NS	NS
**ADMA vs. Tissue mass (g)**	***p *** **< 0.02; R = 0.53**	NS	NS
**ADMA vs. Lean mass (g)**	***p *** **< 0.04; R = 0.49**	NS	NS
**ADMA vs. Fat tissue (g)**	***p *** **< 0.03; R = 0.52**	NS	NS
**ADMA vs. BMI**	NS	NS	***p* < 0.02; R = 0.64**
**VAI vs. HDL**	***p* = 0.002; R = −0.77**	NS	NS
**VAI vs. TG**	***p* = 0.01; R = 0.68**	NS	NS

Abbreviations: **IGF-1**: insulin-like growth factor type 1; **ET-1**: endothelin 1; **ADMA**: asymmetric dimethylarginine; **NO**: nitric oxide;; **TAC**: total antioxidant capacity; **TOC**: total oxidant capacity; **NT-pro-BNP**: N-terminal fragment of the pro brain natriuretic peptide; **Ca**: calcium; **HDL**: high-density lipoprotein; **TG**: triglycerides; **BMC**: bone mineral content; **BMD**: bone mineral density; **BMI**: body mass index; **VAI**: visceral adiposity index.

**Table 4:** Clinical characteristics in the studied group.

Patients (n = 15)	Sex	Age (Years)	Treatment (Before rhGH)	Dose of rhGH	Etiology GHD	IGF-1 (ng/mL) Initially	BMI (kg/m^2^) Initially	CO-GHD in History
**P1**	F	41	HCT, L, D, Es/Pg	0.5 mg	CPGP	68.6	30.9	+
**P2**	M	25	L,T	0.5 mg	NFPM	62.8	24.8	+
**P3**	M	18	T	0.4 mg	CPH	27.3	22.8	+
**P4**	F	26	HCT, L, Es/Pg	0.6 mg	CPH	40.1	29.0	+
**P5**	M	19	D, L, T, HCT	0.3 mg	CPGP	74.8	34.9	+
**P6**	F	60	HCT, L	0.4 mg	ES	15.11	24.3	-
**P7**	M	20	L, HCT, T	0.3 mg	CPH	91.8	28.1	+
**P8**	M	23	-	0.3 mg	I	138.8	25.9	-
**P9**	F	38	L, HCT, Es/Pg	0.5 mg	NFPM	47.07	24.9	-
**P10**	M	18	T	0.2 mg	I	120.2	20.4	+
**P11**	M	28	L, HCT, T, D	0.3 mg	CPGP	22.6	27.1	+
**P12**	M	42	L, T, D	0.3 mg	CPGP	63.0	54.1	+
**P13**	M	36	HCT, L, T	0.5 mg	CPGP	48.9	21.5	+
**P14**	M	18	L, HCT, D, T	0.7 mg	CPGP	54.4	24.4	+
**P15**	M	25	L, HCT, T	0.5 mg	CPGP	8.6	35.8	+

Abbreviations: **GHD**: growth hormone deficiency; **rhGH**: recombinant human growth hormone; **P**: patient; **F**: female; **M**: male; **HCT**: hydrocortisone; **L**: levothyroxine; **Es/Pg**: estrogen/progesterone; **D**: desmopressin; **T**: testosterone; **CPH**: congenital pituitary hypoplasia; **CPGP**: craniopharyngioma postsurgical; **ES**: empty sella; **NFPM**: non-functioning pituitary macroadenoma; **CO-GHD**—childhood-onset growth hormone deficiency; **I**: idiopathic; **IGF-1**: insulin-like growth factor type 1; **BMI**: body mass index.

## Data Availability

The datasets analyzed during the current study are available from the corresponding author upon reasonable request.

## Abbreviations

The following abbreviations are used in this manuscript:

AO-GHD: adult-onset growth hormone deficiency; IGF-1: insulin-like growth factor type 1; ET-1: endothelin 1; ADMA: asymmetric dimethylarginine; NO: nitric oxide; TAC: total antioxidant capacity; TOC: total oxidant capacity; NT-pro-BNP: N-terminal fragment of the pro brain natriuretic peptide Ca: calcium; BMC: bone mineral content; BMD: bone mineral density; VAI: visceral adiposity index; HDL: high-density lipoprotein; TG: triglycerides; BMI: body mass index; GHD: growth hormone deficiency; rhGH: recombinant human growth hormone; P: patient; F: female; M: male; HCT: hydrocortisone; L: levothyroxine; Es/Pg: estrogen/progesterone; D: desmopressin; T: testosterone; CPH: congenital pituitary hypoplasia; CPGP: craniopharyngioma postsurgical; ES: empty sella; NFPM: non-functioning pituitary macroadenoma; CO-GHD: childhood-onset growth hormone deficiency; I: idiopathic.
